# A broadband tunable terahertz negative refractive index metamaterial

**DOI:** 10.1038/s41598-018-28221-3

**Published:** 2018-06-29

**Authors:** Fang Ling, Zheqiang Zhong, Renshuai Huang, Bin Zhang

**Affiliations:** College of Electronics and Information Engineering, Sichuan University, Chengdu, 610065 China

## Abstract

A strategy to greatly broaden negative refractive index (NRI) band, reduce loss and ease bi-anisotropy of NRI metamaterials (MMs) has been proposed at terahertz frequencies. Due to the symmetric structure of the MM, the transmission and refractive index are independent to polarizations of incident radiations, and a broadband NRI is obtainable for the range of the incident angle from 0° to 26°. In addition, THz MMs’ properties such as transmission, phase and negative refraction exhibit a real-time response by controlling the temperature. The results indicate that the maximum bands of the negative and double-negative refraction are 1.66 THz and 1.37 THz for the temperature of 40 °C and 63 °C, respectively. The figure of merit of the MMs exceeds 10 (that is, low loss) as the frequency increases from 2.44 THz to 2.56 THz in the working temperature range, and the maximum figure of merit is 83.77 at 2.01 THz where the refractive index is −2.81 for a given temperature of 40 °C. Furthermore, the negative refraction of the MMs at the low loss band is verified by the classical method of the wedge, and the symmetric slab waveguide based on the proposed MM has many unique properties.

## Introduction

Negative index MMs (NIMs) provide numerous unusual properties and phenomena because of their special interaction with incident radiation, which would be applied to some important fields such as superlens^1^, slow light device^2^, and communication system etc^3^. The first theoretical investigation of NIMs was proposed by Veselago in 1968^4^, and experimentally demonstrated until 2001 by Shelby, Smith and Schultz^5^. Further, the basic mechanism of NIMs condition is *ε*_1_*μ*_2_ + *ε*_2_*μ*_1_ < 0 (the permittivity *ε* = *ε*_1_ + i*ε*_2_, the permeability *μ* = *μ*_1_ + i*μ*_2_) instead of both *ε*_1_ and *μ*_1_ being negative simultaneously^6^. Thus, the NIMs of double-negative MMs with simultaneous negative *ε*_1_ and *μ*_1_ and single-negative MMs with single negative *ε*_1_ or *μ*_1_ can be obtained by tailoring the geometry^7^ or configuring the unit structure arrays^8^. The electromagnetic response of the MMs can be dynamically controlled by changing illumination^9^, temperature^10^ and voltage etc^11^, and the controllable characteristics of the MMs are particularly important for manipulating electromagnetic radiation at terahertz (THz) frequencies where the nature material response is somewhat rare.

Multiphoton microfabrication enables the production of three-dimensional (3D) microstructures with sub-100 nm resolution via direct laser writing (DLW) using a femtosecond laser pulsed^12^, and lithography of 3D polymeric templates by DLW with feature sizes in less than 100 nm has become routine and is even available commercially^13^. Therefore, combined with atomic-layer deposition and/or chemical vapor deposition (CVD), some 3D bulk MMs have been fabricated, especially at frequencies range from mid-IR to visible^13–16^. Accordingly, THz MMs based on three-dimensional 3D standing structures have been fabricated and characterized, and unfortunately, due to the strong bi-anisotropy of the structure, the refractive index is always positive despite of the *ε*_1_ and *μ*_1_ presenting negative values^17,18^. Although the 3D THz NIMs based on standing structure have been achieved, they are sensitive to the polarizations and incident angles, and exhibit a strong bi-anisotropy as well owing to the asymmetric structure^8,19^. Besides, the 3D THz NIMs composed of stacking up multiple fishnet functional layers indicates high figure of merit (FOM)^20–22^, but the NRI band with the FOM higher than 10 is narrow, and the ability to control refractive index is low. Therefore, the realization of waveguide and other applications of these 3D THz NIMs are limited because of the strong bi-anisotropy and the low FOM^23^.

In this paper, a new structure for THz NIMs with symmetric 3D split-ring resonators (SRRs) has been designed and characterized. The NIMs exhibit properties of thermal tunability, broad-band negative refraction, high FOM, polarization independence and low sensitivity to incident angles between 0° and 26°. The performance of the NIMs is stable between 10 °C and 40 °C, and the negative refractive properties can be thermally controlled by tuning the temperature from 40 °C to 80 °C. The maximum bandwidths of the NRI and double-negative index are 1.66 THz and 1.37 THz when the temperature is 40 °C and 63 °C, respectively. Additionally, the maximum FOM is 83.77 for a refractive index of −2.81 at 2.01 THz, and the FOM always exceeds 10 when the frequency increases from 2.44 THz to 2.56 THz at the research range of temperature. Finally, the negative refraction of the NIMs is proved by the classical method of wedge, and applications of symmetric parallel waveguide are also discussed in detail.

## Results and Discussions

**Figure 1:**
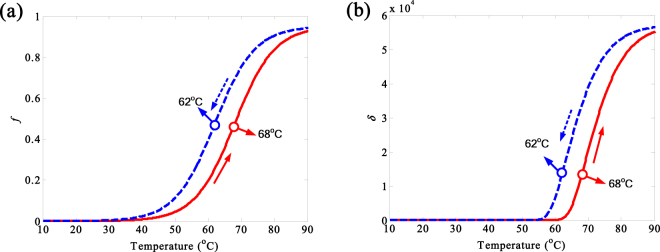
(**a**) The volume fraction *f* and (**b**) the effective conductivity *σ* of VO_2_ at different temperatures for heating (*T*_0_ = 68 °C) and cooling (*T*_0_ = 62 °C) processes with the hysteresis of Δ*T* = 6 °C.

The thermal tunable vanadium dioxide (VO_2_) is an ideal material for smart thermal control system due to its reversible phase transition from an insulator to a metallic phase at critical temperature of around 67 °C^24^, and the phase transition time is on a scale of 10 ns by heating^25^, as well as its effective conductivity. Another important feature is that VO_2_ is highly transparent for electromagnetic wave below 6.7 THz^26^. The dielectric function *ε*_*m*_ of metallic phase follows the Drude^27^, i.e.

1 $${\varepsilon }_{m}(\omega )={\varepsilon }_{\infty }-\,\frac{{\omega }_{p}^{2}}{{\omega }^{2}+i\omega /\tau }$$

where the *ε*_∞_ = 9 represents the high-frequency permittivity; *ω* denotes angular frequency of THz radiation. *τ* = m^*^μ/*e* is the relaxation time, the m^*^ = 2*m*_*e*_ represents the effective mass, and the carrier mobility is *μ* = 2 cm^2^/(V·s). The plasma frequency is *ω*_*p*_ = (*Ne*^2^/(*ε*_0_m^*^))^−1/2^, the effective mass carrier density is *N* = 8.7 × 10^21^ cm^−3^, *m*_*e*_ and *e* represent the mass of free electron and electronic charge, respectively. The effective dielectric function of VO_2_ film in the process of insulator-metal transition can be well described by the Bruggeman effective medium theory (EMT)^28^:

2 $${\varepsilon }_{{\rm{eff}}}=\frac{1}{4}\{{\varepsilon }_{i}(2-3f)+{\varepsilon }_{m}(3f-1)+\sqrt{{[{\varepsilon }_{i}(2-3f)+{\varepsilon }_{m}(3f-1]}^{2}+8{\varepsilon }_{i}{\varepsilon }_{m}}\}$$

where *ε*_*i*_ is insulating permittivity, and *ε*_*i*_ = *ε*_∞_ = 9. The volume fraction *f* of conductive regions is given by^28^:

3 $$f(T)={f}_{\max }\{1-\frac{1}{1+\exp [(T-{T}_{0})/{\rm{\Delta }}T]}\}$$

where *T*_0_ denotes the transmission temperature of VO_2_ (the heating transmission temperature *T*_0_ = 68 °C, the cooling transmission temperature *T*_0_ = 62 °C); the hysteresis is Δ*T* = 6 °C; the volume fraction *f*_max_ of metallic domain is 0.95. The effective conductivity *σ* of the metallic phase VO_2_ can be described as^29^:

4 $$\sigma =i{\varepsilon }_{0}\omega (1-{\varepsilon }_{eff})$$

where *ε*_0_ labels vacuum permittivity. According to the Eqs (1–4), the thermal properties of the VO_2_ can be simulated, as given in Fig. 1.

It is clear that the volume fraction *f* and the effective conductivity *σ* of VO_2_ varying with the temperature show thermal tunability and a typical hysteresis behavior. During the heating process, *f* remains below 1% (i.e., the metallic property of VO_2_ can be ignored) at the temperature between 10 °C and 40 °C. Although *f* increases obviously with the temperature raising from 40 °C to 60 °C, the effective conductivity *σ* is below 100 S/m. When the temperature rises from 61 °C to 80 °C, *σ* increases abruptly from 173.5 S/m to 4.673 × 10^4^ S/m due to the metallic phase transition at 68 °C. If the temperature exceeds 80 °C, *σ* stabilizes gradually. Similarly, *σ* represents thermal tunability as well for the cooling process, while the critical temperature changes to 62 °C. Accordingly, the THz thermal tunable 3D NIMs composed of quad-vertical metallic SRRs and thermo-sensitive VO_2_ films integrated in the substrate has been proposed. In order to enhance the magnetic response and ease bi-anisotropy of the MM, the symmetric metal slices on the front and bottom surfaces of the dielectric substrate are connected via metal cylinders.

**Figure 2:**
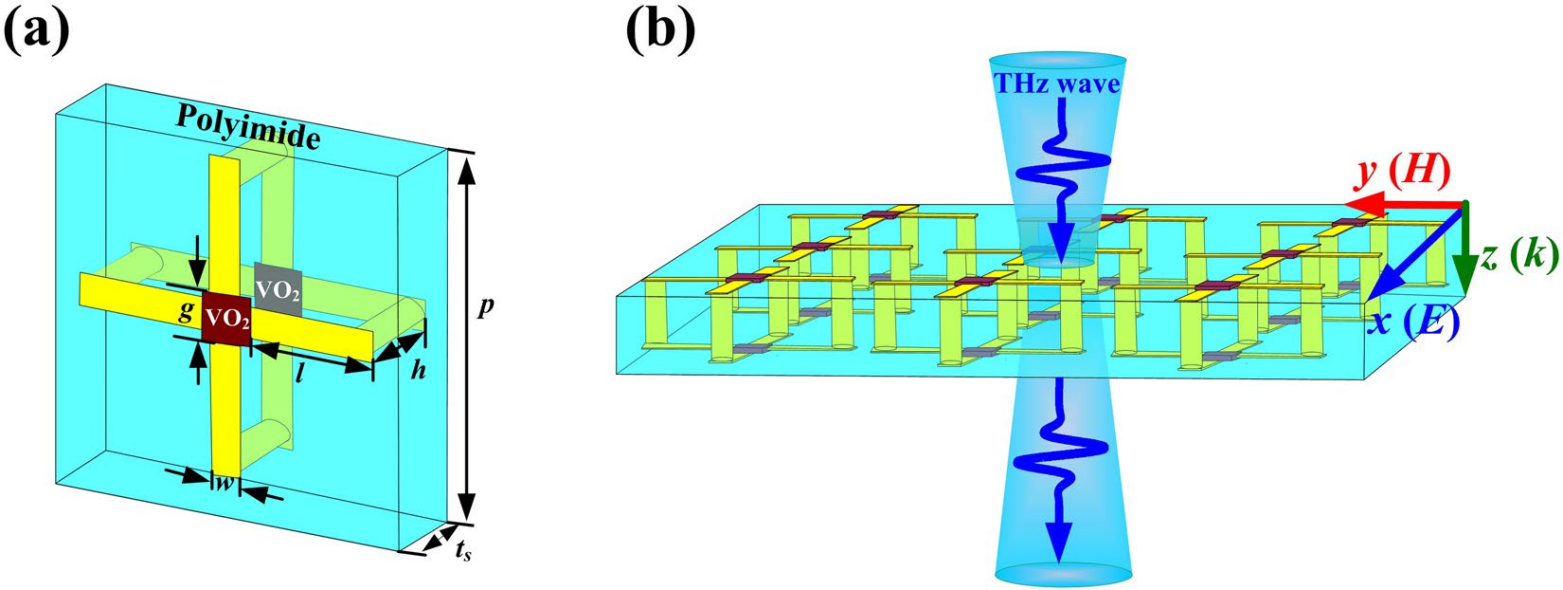
Schematic of the broadband THz NIMs. (**a**) Typical dimensions for unit cell of the SRRs. (**b**) Perspective view of the NIMs array at the normally incident THz wave.

Figure 2 details the structure of the THz NIMs with the lateral dimension *p* = 70 μm, and the length (*l*) and the width (*w*) of the SRRs are 25 μm and 6 μm, respectively. The thicknesses of the metallic portions, VO_2_ films and dielectric spacer are *t*_*c*_ = 0.2 μm, *t*_*v*_ = 0.5 μm and *t*_*s*_ = 18 μm, respectively. The heights of the vertical cylinder (*h*) are same as *t*_*s*_, and the radius of the vertical cylinder (*r*) and the gaps of the SRRs (*g*) are 3 μm and 10 μm, respectively. Generally, the geometries of VO_2_ films with less than 10 μm can be shaped by lithography and etching^30^, and there are many the hybrid structures composed of metallic resonators and VO_2_ films have been fabricated and reported^31–33^. Commonly, the first fabrication step of these hybrid structures is shaping the VO_2_ film by combing the lithography and etching technology, and the second step is fabricating the metallic resonators. Therefore, the THz 3D structure composed of metallic resonators and square VO_2_ film with length 10 μm × 10 μm and gap width 60 μm × 60 μm can be fabricated as well. The first step is shaping the square VO_2_ films by lithography and etching^30–33^, and the second step is fabricating the inherent connective cylinders by DLW and CVD^13–16,34,35^.

The transmission of the THz NIMs is calculated by the commercial program CST Microwave Studio 2014 with the frequency domain solver. The propagation wave vector (***k***) is perpendicular to the NIMs whereas the electric field (***E***) and magnetic field (***H***) are parallel to the incident plane (floquet ports are set along the *z* direction, and unit-cell boundaries are applied to the *x* and *y* directions). The tetrahedral meshes with the adaptive meshing method are employed. The lossy copper with a frequency-independent conductivity *σ* = 5.8 × 10^7^ S/m^36^ and the lossy polyimide with a relative permittivity *ε*_*r*_ = 3.5^37^ are chosen as the metallic pattern and the dielectric spacer to accurately calculate the 3D MMs, respectively.

**Figure 3:**
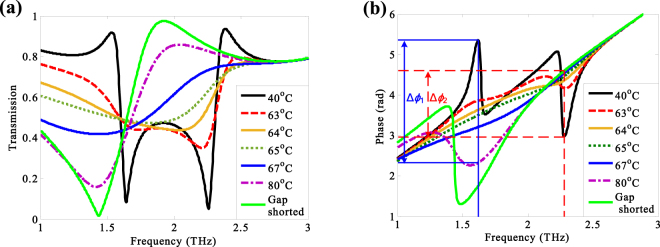
(**a**) The normalized transmission spectra and (**b**) transmission phase spectra at different temperatures. The green-solid curves correspond to the gaps shorted with copper. The blue-solid and red-dashed lines of (**b**) indicate the phase shifts at 1.62 THz and 2.28 THz, respectively.

The normalized transmission and phase spectra of the THz NIMs at different temperatures are plotted in Fig. 3. The performance of the NIMs is stable with the temperature increasing from 10 °C to 40 °C due to the almost unchanged conductivity of the VO_2_ films [see the Fig. 1]. As given in Fig. 3(a), there are two resonance frequencies at 1.64 THz and 2.23 THz for a given temperature of 40 °C. With the temperature increasing from 40 °C to 63 °C, the lower resonance frequency presents blue-shift, while the higher resonance frequency exhibits red-shift. The lower resonance frequency disappears, and the higher resonance frequency decreases continuously when the temperature increases to 64 °C. The resonance frequency redshifts to 1.83 THz with the temperature increasing to 65 °C. As the temperature reaches to 67 °C, the resonance frequency further shifts to 1.42 THz with a transmission of 41.79%. Further, the transmission dip located at 1.42 THz decreases to 15.96% when the temperature increases to 80 °C. The gaps are totally shortened by copper, and therefore, the transmission dip locates at 1.44 THz with a transmission dip of 1.59% owing to the conductivity of copper higher than that of the VO_2_ films.

Another functionality of the THz NIMs manipulated by temperature is phase shifting. As the blue-solid vertical line indicated in Fig. 3(b), the phases of the THz transmission are *ϕ*_40_ = 5.35 rad and *ϕ*_80_ = 2.33 rad at 1.62 THz, and the corresponding phase shift is Δ*ϕ*_1_ = 0.96π. Meanwhile, indicated by the red-dashed vertical line, the phases of the THz transmission are *ϕ*_40_ = 2.79 rad and *ϕ*_80_ = 4.60 rad, resulting in a phase shift of Δ*ϕ*_2_ = −0.58π at 2.28 THz. Hence, the designed NIMs can function as a THz double-phase shifter. In order to study and understand the dynamic coupling effect of the NIMs for different temperatures, the distributions of the surface current at different resonance frequencies are calculated.

**Figure 4:**
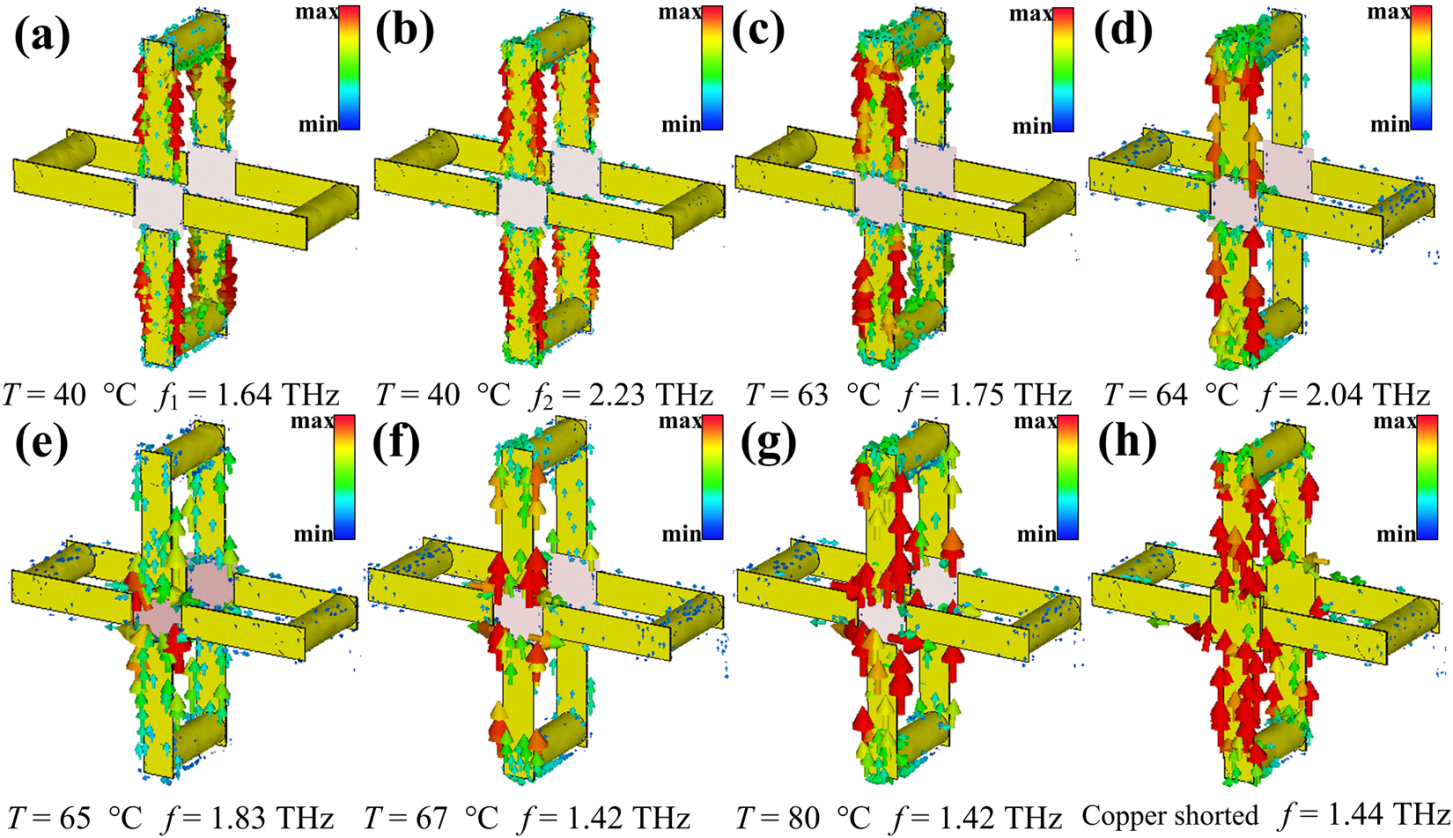
The surface current distributions of the SRRs normally incident by THz radiation for a given temperature and resonance frequency.

Figure 4(a,b) presents the surface current distributions of the SRRs corresponding to the two resonance frequencies for a given temperature *T* = 40 °C. At the lower resonance frequency (*f*_1_ = 1.64 THz), the circulating current causes a lot of charges accumulation on both gaps forming an inductive–capacitive (*LC*) resonance, which can be considered as a magnetic dipole described by Lorentz model^38^. At the higher resonance frequency (*f*_2_ = 2.23 THz), the surface current exhibits symmetric parallel loops on the top and bottom arms, which gives rise to a strong scattering leading to a broad dipole resonance feature^39^. It can be seen in Fig. 4(c) that the density of the surface current decreases with the temperature rising to 63 °C, which leads to the attenuation of the *LC* resonance. From Fig. 4(d) we can see that the surface current at 2.04 THz focuses on the top arm of the SRRs for the case of temperature *T* = 64 °C. Therefore, the *LC* resonance disappears, and the gaps of the SRRs are effectively shorted by VO_2_ films.

**Figure 5:**
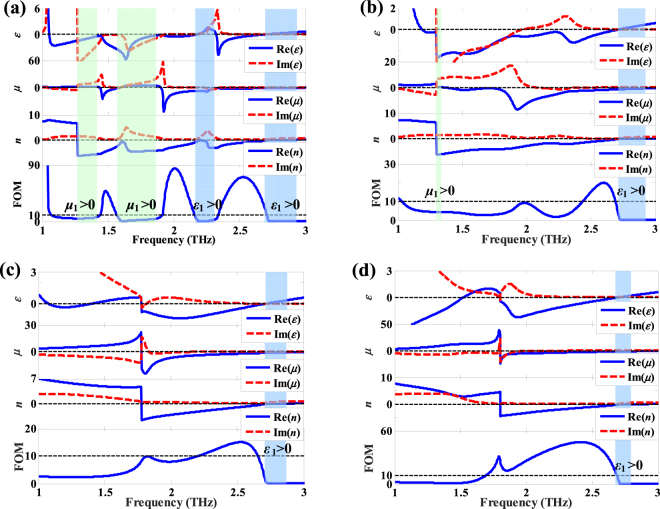
The retrieved parameters of the NIMs for a given temperature (**a**) 40 °C, (**b**) 63 °C, (**c**) 67 °C and (**d**) 80 °C, respectively. The green and blue regions mark the ranges of single-negative index with *ε*_1_ > 0 and *μ*_1_ > 0, respectively.

In Fig. 4(e), the symmetric parallel current distribution is observed. Figure 4(e–g) depicts that the resonance is gradually strengthened with the increasing temperature from 65 °C to 80 °C. For the case of the gaps shorted by copper, the density of the surface current increases further, as illustrated in Fig. 4(h). Consequently, the reasons why the blueshift of the *LC* resonance frequency and the redshift of the dipole resonance frequency can be regarded as the decreasing effective *C* and the increasing effective *L* (*f*_resonance_ ∝ 1/(*LC*)^−1/2)^^40,41^, respectively. In order to study the characteristics of the NIMs, Fig. 5 gives the retrieved constitutive parameters of the NIMs at different temperatures.

Because the scale of the unit cell of the NIMs is smaller than the operating wavelength and the structure is symmetric along the THz propagation, i.e. *S*_11_ = *S*_22_, the standard retrieval method is performed to extract the effective *ε* and *μ*^42^. The figure of merit (FOM, FOM = |Re(*n*)/Im(*n*)|) is a well-established measure of the strength of the NRI with respect to the losses^20,43,44^. Figure 5 shows the retrieval parameters and FOM as a function of frequency at the normal incident angle with different temperatures, in which the green and blue regions reveal the ranges of single-negative refraction with positive *μ*_1_ and *ε*_1_, respectively. In Fig. 5(a), there is a broadband of negative refraction that lies between 1.27 THz and 2.93 THz when the temperature is *T* = 40 °C, and the broadband is composed of four single-negative and three double-negative bands which are highlighted by the green and blue areas, respectively. Additionally, the FOM in double-negative region is greater than that in single negative regions, and the maximum value of FOM is 83.77 at 2.01 THz (where Re(*n*) is −2.81). For the case of *T* = 63 °C shown in Fig. 5(b), the maximum bandwidth of the double-negative refraction is obtained, which lies between 1.35 THz and 2.72 THz. However, the FOM in the double-negative refraction region decreases with the weakening resonance.

For the cases of *T* = 67 °C and *T* = 80 °C depicted in Fig. 5(c,d), the negative-refractive band of the NIMs only has a single-negative refraction band with *ε*_1_ > 0 at the higher frequency. The FOM of the double-negative refraction increases with the increasing temperature from 63 °C to 80 °C, because the resonance of the NIMs is gradually enhanced with the increasing conductivity of the VO_2_ films. Consequently, the tunable NRI is achieved, and the maximum bandwidths of the NRI and double NRI are 1.66 THz and 1.37 THz for the temperature of 40 °C and 63 °C, respectively. The FOM of the double-negative band between 2.44 THz and 2.56 THz is always higher than 10 (that is, low loss) when the temperature increases from 40 °C to 80 °C.

**Figure 6:**
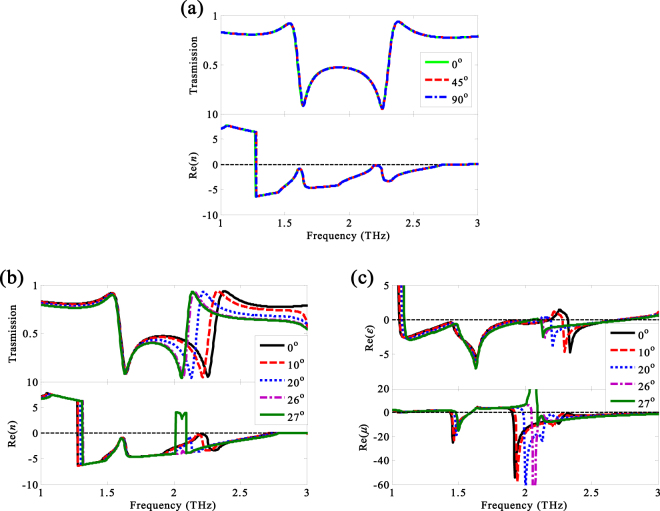
The normalized transmission spectra and refractive index of the NIMs (**a**) at different polarization angles and (**b**) different incident angles for a given temperature *T* = 40 °C. (**c**) The real parts of the permittivity and permeability of the NIMs at different incident angles when the temperature is *T* = 40 °C.

In practical applications such as THz waveguide, the independence of polarization and incident angles is desirable. Hence, the impacts of polarization and incident angels on the transmission and refraction also are shown in Fig. 6.

Due to the symmetrical characteristic of the NIMs, the normalized transmission and the real part of the refractive index of the NIMs are independent of polarization angles under the normally incident THz radiation for the case of temperature *T* = 40 °C, as given in Fig. 6(a). From Fig. 6(b) we can see that the transmission and the real part of the refractive index of the NIMs have low sensitivity to the incident angles between 0° and 26°, whereas a positive refractive index band appears in the NRI band at 27°. The transmission and refraction at the low frequency change slightly with the increasing incident angle, but change drastically at high frequency. In order to further study the electromagnetic response of the NIMs at different incident angles, Fig. 6(c) gives the real parts of *ε*_1_ and *μ*_1_ of the NIMs at different incident angles. The positive refractive index band is induced by the magnetic response instead of the electric response at 27°. Consequently, a tunable THz NIMs with the properties such as thermo tunability, polarization independence, less incident sensitivity and high FOM is realized.

A popular and useful way to demonstrate the negative refraction is to calculate a wedge^38^. The wedge samples of the NIMs are composed of 6 × 6 unit cells in both *x* and *z* directions with one-unit-cell stairs. The adjacent unit cells are contact in *x* direction, and one unit cell is considered in *y* direction because the MMs in *x* and *y* directions are symmetric and periodic. Thus, it is not necessary to construct the negative refractive behavior in y direction. Besides, in order to avoid the interference between the adjacent unit cells in propagation direction (*z* direction), the adjacent samples are separated by vacuum with thickness of 2 μm. Since the periodicities along the *x* and *z* directions are *p*_*x*_ = 70 μm and *p*_*z*_ = 19.4 μm, the incident angle is determined as *θ*_*i*_ = arctan (*p*_*z*_/*p*_*x*_) ≈ 15.49°.

**Figure 7:**
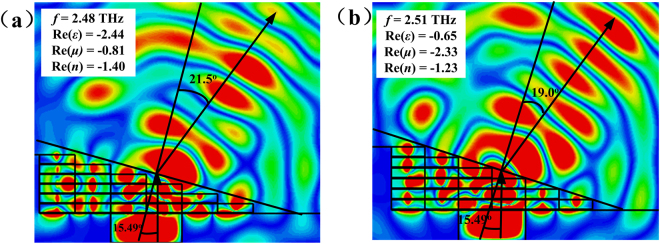
The electric field distributions of the refracted THz radiation at (**a**) 2.48 THz and (**b**) 2.51 THz for the NIMs at 40 °C.

Figure 7 plots the electric field distributions of the wedge at 2.48 THz and 2.51 THz in the *y* plane for a given temperature *T* = 40 °C. According to the wave fronts indicated by localized field with the strong intensity, the refracted angles *θ*_*r*_ of the wedge sample are about 21.5° and 19.0° at 2.48 THz and 2.51 THz, respectively. By utilizing the Snell’s law *n* = sin *θ*_*r*_/sin *θ*_*i*_, the real parts of the refractive indexes Re(*n*) of the NIMs at 2.48 THz and 2.51 THz are estimated as −1.40 and −1.22, respectively. The retrieval Re(*n*) of the NIMs at 2.48 THz and 2.51 THz are −1.40 and −1.23, which indicates that the retrieval values of the Re(*n*) are consistent with that estimated by the Snell’s law of the wedge.

The waveguide composed of high FOM NIMs, which is an important device of the THz transmission system, has many unique properties^45^ such as lack of fundamental mode and cutoff frequency. Accordingly, the thermally controlled NIMs at the low loss band can be applied to the symmetric slab waveguide. The *k*_1*x*_ and *k*_2*x*_ are the transverse wave numbers in free space and NIMs along the *x* direction, respectively, and can be written as^45,46^:

5 $${k}_{1x}^{2}={\beta }^{2}-\frac{{\omega }^{2}}{{c}^{2}}{\varepsilon }_{1}{\mu }_{1}$$

6 $${k}_{2x}^{2}=\frac{{\omega }^{2}}{{c}^{2}}{\varepsilon }_{2}{\mu }_{2}-{\beta }^{2}$$

where Re(*β*) is the THz wave transmission constant, the *ω* is the angular frequency of the incident field. The guidance condition of the homogeneous NIMs symmetric slab waveguide can be written as:

7 $${k}_{1}d=\frac{{\mu }_{1}}{{\mu }_{2}}{k}_{2x}d\cdot \,\tan (\frac{{k}_{2x}d}{2}-\frac{m\pi }{2})$$

therefore, for even *m*, the guidance condition can be described as:

8 $${k}_{1x}d=\frac{{\mu }_{1}}{{\mu }_{2}}{k}_{2x}d\cdot \,\tan (\frac{{k}_{2x}d}{2})$$

for the odd *m* mode, Eq. (7) yields

9 $${k}_{1x}d=-\,\frac{{\mu }_{1}}{{\mu }_{2}}{k}_{2x}d\cdot \,\cot (\frac{{k}_{2x}d}{2})$$

according to the dispersion relations between the different regions:

10 $$\,\{\begin{array}{l}{({{k}}_{{z}}{d})}^{2}-{({{k}}_{1{x}}{d})}^{2}={({{k}}_{1}{d})}^{2}\\ {({{k}}_{{z}}{d})}^{2}+{({{k}}_{2{x}}{d})}^{2}={({{k}}_{{\rm{2}}}{d})}^{2}.\end{array}$$

**Figure 8:**
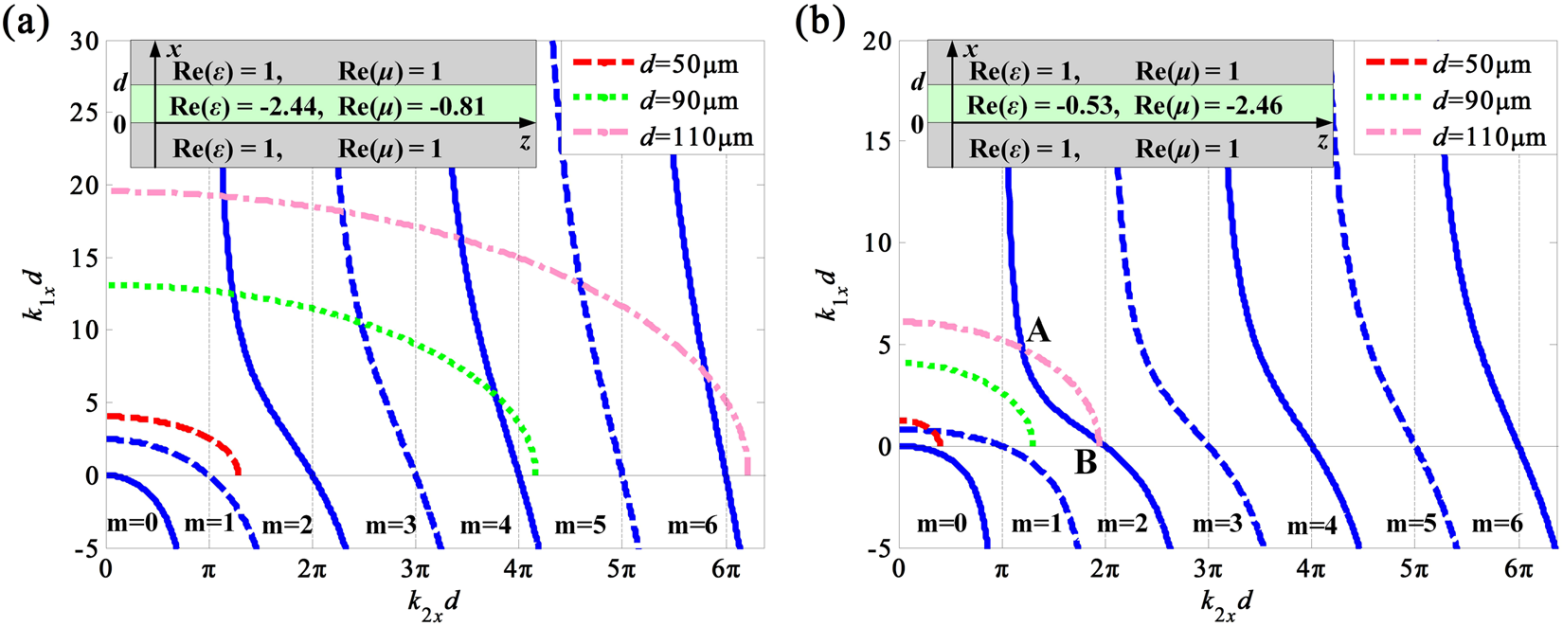
The TE guided modes of the NIMs symmetric slab waveguide with different thicknesses at 2.48 THz for the given temperatures of (**a**) 40 °C and (**b**) 80 °C. The insets of (**a**) and (**b**) are the schematics of the NIMs waveguide geometries, and the *z* direction is chosen as the direction of propagation of guided modes. The A and B points in (**b**) represent that there are two different intersections.

The circle function *ρ* = (*k*_1*x*_*d*)^2^ + (*k*_2*x*_*d*)^2^ can be defined according to the electromagnetic parameters of media 1 and 2. In Eq. (10), *k*_*z*_ denotes the transverse wave number in *z* direction, *k*_1_ and *k*_2_ are the transverse wave numbers in free space and NIMs, respectively. Thus, the transcendental Eqs (8, 9) can be solved by the graphical method. Accordingly, Fig. 8 depicts that the transverse electric (TE) guided modes of the symmetric slab waveguides with different thicknesses *d* are calculated for a given temperature of 40 °C and 80 °C at 2.48 THz, respectively.

The schematics of the NIMs symmetric slab waveguides with different parameters of NIMs in free space are given in the insets of Fig. 8, and the gray and green areas indicate the free space and NIMs, respectively. The solid-blue curves present the even mode with different *m*, and dashed-blue curves are odd mode with different *m*, respectively. The red-dashed, green-dotted and pink-dashed-dotted curves are *ρ* = (*k*_1*x*_*d*)^2^ + (*k*_2*x*_*d*)^2^ with different values *d*. The intersections of the curves indicate the existence of solutions for guided modes^45^. It can be seen that the NIMs slab waveguide do not support any TE wave at 40 °C, while the TE_1_ mode is supported at 80 °C when *d* is 50 μm. For *d* = 90 μm, the NIMs slab wave guide supports TE_2_, TE_3_ and TE_4_ modes simultaneously for a given temperature of 40 °C, whereas the NIMs waveguide do not support any TE wave at 80 °C. If *d* increases to 110 μm, the TE_2_, TE_3_, TE_4_, TE_5_ and TE_6_ modes are supported for the temperature of 40 °C. Besides, there are two modes co-existing for the TE_2_ mode, as A and B points represented in Fig. 8(b), which is an unique performance of NIMs waveguide.

Consequently, the performance of the NIMs waveguide can be dynamically tuned by controlling temperature between 40 °C and 80 °C at the low loss frequency band. Moreover, the NIMs has many unique properties different from the conventional slab waveguide: firstly, there is no fundamental guided mode (that is, TE_0_ mode) no matter what the value of *d* is, because the transvers wave number *k*_1*x*_ is negative, while the *ρ* is always positive^45^. Secondly, for *m* = 1, the transvers wave number *k*_1*x*_*d* is a determined value for *k*_2*x*_*d* = 0, and *k*_1*x*_*d* is 0 for *k*_2*x*_*d* = π, as described in Eq. (9) and Fig. 8. Therefore, the lowest mode TE_1_ of the NIMs waveguide cannot be arbitrary value from 0 to +∞ (that is, the TE_1_ mode has cutoff frequency), while the lowest mode of the conventional slab waveguide has no cutoff frequency. In addition, the two modes may be co-existing for a mode, as A and B intersection illustrated in Fig. 8(b).

## Conclusion

A THz NIMs composed of symmetric SRRs and thermo-sensitive VO_2_ films has been proposed. The transmission, phase and refractive index of the proposed NIMs are dynamically tunable with the increasing temperature from 40 °C to 80 °C. Due to the symmetric structure of the NIMs, the transmission and refractive index are independent to the polarizations of the incident THz radiation, and exhibits low sensitivity to the incident angles between 0° to 26°. When the frequency rises from 2.44 THz to 2.56 THz, the FOMs always exceed 10, and its peak value is 83.77 for the case of *f* = 2.01 THz, *T* = 40 °C. The negative refractive characteristics of the NIMs at 2.48 THz and 2.51 THz are verified by the classical method of the wedge, which coincides with the retrieval values of the NRI. Besides, the NIMs symmetric slab waveguide has many unique properties such as lack of fundamental mode, cutoff frequency and co-existing of two modes. The properties of the NIMs slab waveguide are dynamically tunable by controlling the temperature from 40 °C to 80 °C, whereas they are in steady-state when the temperature increases from 10 °C to 40 °C.
